# Liver hydatid cyst rupture into gallbladder and right hepatic duct: A case report

**DOI:** 10.1016/j.ijscr.2024.110305

**Published:** 2024-09-18

**Authors:** Mohammad Samim Fahmi, Wais Farda, Zabihullah Sharif, Rana Sarhadi Jamal

**Affiliations:** a Chief General Surgery Department, General Surgery Department, Cure Hospital, Darulaman Road, Kabul, Afghanistan; b Consultant Surgeon and Associate Clinical Professor, General Surgery Department, Isteqlal Hospital, Alaudin Square, Darulaman Road, Kabul, Afghanistan; c Attending General Surgeon, General Surgery Department, Cure Hospital, Darulaman Road, Kabul, Afghanistan; d General Surgeon, General Surgery Department, Cure Hospital, Darulaman Road, Kabul, Afghanistan

**Keywords:** Hydatid cyst, Gallbladder, Biliary tract, Rupture

## Abstract

**Introduction and importance:**

Concomitant rupture into the gallbladder and biliary tract is a very rare complication of liver hydatid cyst.

**Presentation of case:**

We present the case of a young woman with rupture of liver hydatid cyst into gallbladder and right hepatic duct which was presented with abdominal pain and jaundice.

**Clinical discussion:**

While fistulization to the gallbladder is a rare complication of liver hydatid cyst, concomitant rupture of liver hydatid cyst into the gallbladder and biliary tract is very rare. The principle treatment of this condition is surgery with adjuvant anthelmintic therapy.

**Conclusion:**

Rupture into gallbladder and biliary tract is a rare complication of liver hydatid cyst, where abdominal pain is the most common symptom and surgery is the mainstay of treatment.

## Highlights

• Concomitant rupture into the gallbladder and biliary tract is a very rare complication of liver hydatid cyst.
• Increased intracystic pressure along with inflammation are thought to cause necrosis and lead to fistulization.
• The principle treatment of this condition is surgery with adjuvant antihelmentic therapy.

## 1 Introduction and importance

Liver hydatid cyst complications are not uncommon with the reported incidence from 30 to 60 % in various studies. The most common complications of the liver hydatid cyst are super-infection and intrabiliary rupture, followed by rupture to peritoneal and pleural cavity, while rupture to gastrointestinal tract or other viscera are rare [1,2]. Concomitant rupture of liver hydatid cyst into the gallbladder and biliary tract is very rare and was reported by Wani et al. [3].

We report a case of liver hydatid cyst rupture into the gallbladder and intrahepatic part of right hepatic duct causing obstructive jaundice. This case has been reported in line with the SCARE criteria [4].

## 2 Presentation of case

A 37 year old lady presented to the hospital complaining of right upper abdominal pain for 2 months. She also complained of mild yellow skin discoloration for 1.5 months. The patient denied having any nausea/vomiting, diarrhea or constipation. Past medical/surgical, family and social history were unremarkable. Vital signs were in normal range.

**Fig. 1:**
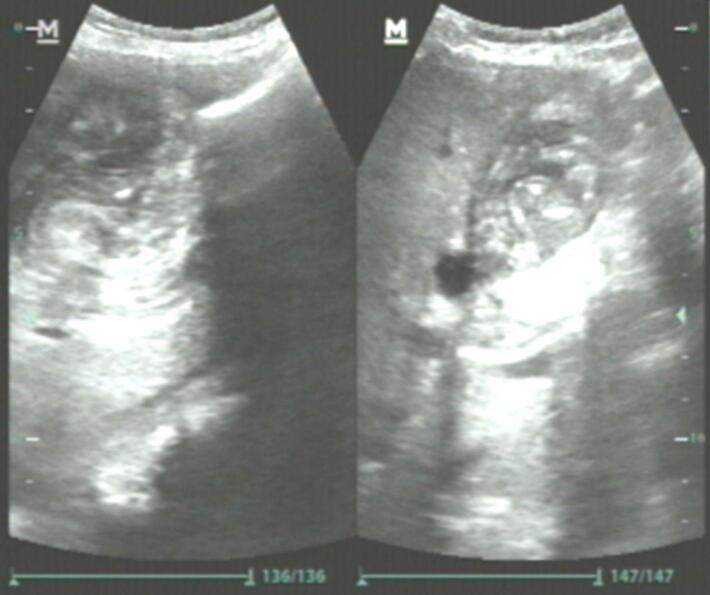
Ultrasound showing multiple linear echogenic structures in gallbladder.

Physical examination revealed tenderness over right upper quadrant of abdomen, with yellowish discoloration of skin and sclera. Laboratory tests showed ALT – 108 u/l, AST – 162 u/l, Alkaline phosphatase – 246 u/l, total bilirubin – 7.5 mg/dl (direct – 5.4 mg/dl, indirect – 2.1 mg/dl), BUN – 31 mg/dl, Creatinine – 0.8 mg/dl. Hepatitis A, B, and C were negative. Stool exam showed ascaris worm ova. Abdominal ultrasound showed dilated intrahepatic biliary ducts and common hepatic duct with multiple linear echogenic structures in the lumen of common bile duct and gallbladder, suggestive of Ascaris worms (Fig. 1).

The patient was diagnosed to have obstructive jaundice and advised admission for surgical intervention, but the patient refused surgical intervention at that time saying she needed time to think and consult with her family. However, she presented again to the hospital two weeks later complaining of increased right upper quadrant pain, increased jaundice, body itching, chills with low grade fever. Physical exam revealed yellow sclera and skin with right upper quadrant tenderness. The vital signs were in normal rage except for tachycardia. The laboratory tests were repeated with the following results: Total bilirubine 9.9 mg/dl, ALT 85 u/l, AST 90 u/l, GGT 54 u/l, alkaline phosphatase 771 u/l, Creatinine 0.7 mg/dl. Abdominal ultrasound was repeated which revealed the same finding as previous imaging. The patient refused CT scan and ERCP due to economic issues.

**Fig. 2:**
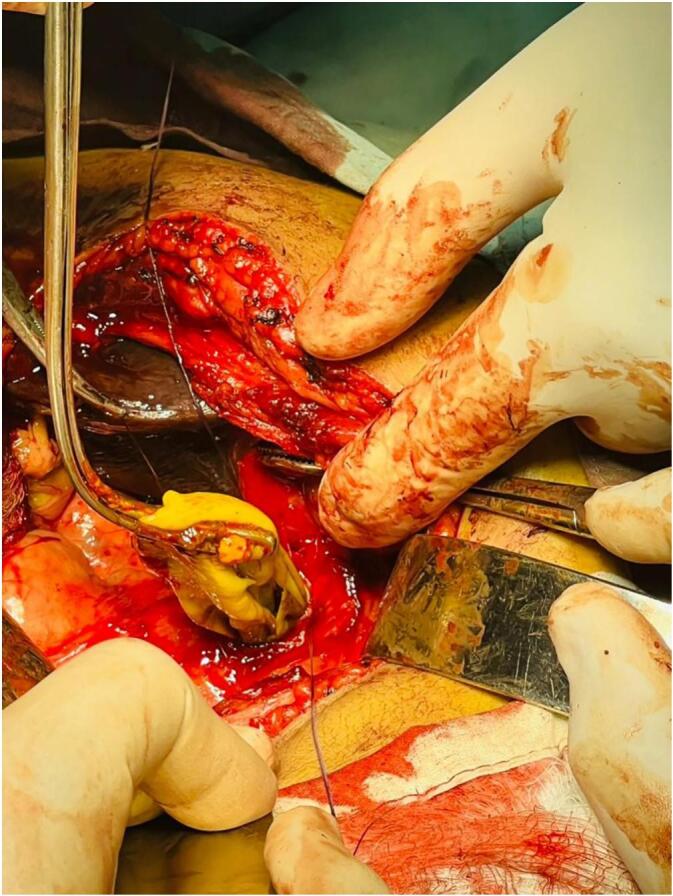
Laminated membrane being pulled-out from choledochotomy.

The patient was admitted in surgical ward and planned for surgery the next day. Abdomen was opened with a Kocher's right subcostal incision. Gallbladder and proximal CBD were distended and full of solid material. A choledochotomy was done and debris of different sizes as well as a big laminated membrane like structure were seen inside the CBD which were evacuated (Fig. 2); at this time the gallbladder became empty. Upon dissecting the gallbladder from the liver, a ∼3.5 cm fistulous opening was found between the gallbladder and its bed in the liver with part of the laminated membrane hanging in the gallbladder. The fistula was connected with the right hepatic duct. After removal of the gallbladder, the fistula was repaired and 18Fr T-tube was placed in the CBD. Abdomen was closed in usual way and a tube drain was placed in subhepatic space. The post-operative period was uneventful, the subhepatic tube drain was removed 48 h post-operative when there was no discharge from the drain and no clinical or ultrasound evidence of any fluid in peritoneal cavity. The patient was discharged on 5th post-operative day with Albendazole therapy regime (15 mg/kg daily in two divided doses for two months) after consulting with internal medicine consultant. The T-tube was removed two weeks later when the patient came for follow-up; the patient did not have any complain, the yellow discoloration of the sclera and the skin was gone and liver enzymes were within normal range. The patient was followed-up after 6 months by phone and she did not report any complain or complication.

## 3 Clinical discussion

Liver hydatid cyst complications are not uncommon with the reported incidence from 30 to 60 % in various studies. The most common complications of the liver hydatid cyst are superinfection and intrabiliary rupture, followed by rupture into peritoneal and pleural cavity, while rupture to GI tract or other viscera are rare [1,2].

Although cases of primary hydatid cyst of the gallbladder have been reported, secondary hydatid cysts of the gallbladder are thought to have resulted from intrabiliary rupture of the cysts [5,6]. Intrabiliary rupture can be frank and cause obstructive jaundice, or may be occult and manifest itself as a biliary fistula. Increased intracystic pressure along with inflammation are thought to cause necrosis and lead to fistulization [7,8].

While fistulization to the gallbladder is a rare complication of liver hydatid cyst with few reported cases in the literature [[8], [9], [10], [11]], concomitant rupture of liver hydatid cyst into the gallbladder and biliary tract is very rare and was reported by Wani et al. [3]. Fistulization between liver hydatid cyst, gallbladder and intestinal tract has also been reported [12,13]. In our case the liver hydatid cyst had ruptured concomitantly into the gallbladder and intrahepatic part of right hepatic duct.

Abdominal pain is the most common symptom of gallbladder hydatid cysts, which can be the only symptom in some cases, while other symptoms including nausea/vomiting, fever and jaundice may also be present [5,6]. Rupture of the hydatid cyst to the gallbladder may present as cholangitis [14,15]. Our case presented with abdominal pain and jaundice.

Abdominal ultrasound and CT scan are the most commonly used imaging modalities in liver hydatid cyst cases, while MRI and MRCP are useful in cases where ultrasound and CT scan findings are inconclusive [16]. Although serological test (IgG antibodies) and ELISA are widely available for hydatid disease, negative results do not exclude presence of hydatid cyst in the body. The positivity of ELISA assays is said to increase with duration of the infestation and age of the patient. Therefore, the serological tests alone are insufficient for definitive diagnosis. There is no serological test to differentiate between subspecies of Echinococcus [17,18].

Surgical options for liver hydatid cysts are divided into conservative (parenchyma preserving) and radical (liver resection) procedures. The conservative procedures include deroofing of the cyst with evacuating and removing the endocyst. The main disadvantage of conservative procedures is high recurrence rate of 8–20 %. Radical procedures include liver anatomical resections like segmentectomy or non-anatomical resections e.g. cystopericystectomy. Radical procedures have the advantage of lowering local recurrence and less biliary leakages [18]. The mainstay treatment of gallbladder hydatid cysts is surgery, which includes cholecystectomy along with dealing with hydatid cyst and the fistula. Anthelmintic therapy with albendazole or mebendazole is also suggested. In our case cholecystectomy with CBD exploration and T-tube drainage was performed and patient was discharged with albendazole therapy regime.

## 4 Conclusion

Rupture into gallbladder and biliary tract is a rare complication of liver hydatid cyst, where abdominal pain is the most common symptom and surgery is the mainstay of treatment. Timely diagnosis and management are key to successful treatment.

## Abbreviations

ALT: alanine transaminase
AST: aspartate aminotransferase
BUN: blood urea nitrogen
CBD: common bile duct
CT: computed tomography
ELISA: enzyme-inked immunosorbent assay
GGT: gamma-glutamyl transferase
IgG: IMMUNOGLOBULIN G
MRI: magnetic resonance imaging
MRCP: magnetic resonance cholangiopancreatography

## Consent

Written informed consent was obtained from the patient for publication of this case report and accompanying images. A copy of the written consent is available for review by the Editor-in-Chief of this journal on request.

## Provenance and peer review

Not commissioned, externally peer-reviewed.

## Ethical approval

This study was approved by the Ethics Committee of the hospital.

## Funding

None.

## Author contribution

Mohammad Samim Fahmi, Wais Farda, Zabibullah Sharif and Rana Sarhadi Jamal: Conceptualization, writing – original draft. Wais Farda: writing – review and editing. All authors read and approved the final manuscript.

## Guarantor

Wais Farda and Mohammad Samim Fahmi.

## Research registration number

Not applicable.

## Conflict of interest statement

None.
